# On the mechanism behind the inverse melting in systems with competing interactions

**DOI:** 10.1038/s41598-018-38465-8

**Published:** 2019-02-14

**Authors:** Alejandro Mendoza-Coto, Lucas Nicolao, Rogelio Díaz-Méndez

**Affiliations:** 1 Departamento de Física, Universidade Federal de Santa Catarina, 88040-900 Florianópolis, Brazil; 2 Department of Theoretical Physics, KTH Royal Institute of Technology, SE-106 91 Stockholm, Sweden

## Abstract

The competition between a short range attractive interaction and a nonlocal repulsive interaction promote the appearance of modulated phases. In this work we present the microscopic mechanisms leading to the emergence of inverse transitions in such systems by considering a thorough mean-field analysis of a variety of minimal models with different competing interactions. We identify the specific connections between the characteristic energy of the homogeneous and modulated phases and the observed reentrant behaviors in the phase diagram. In particular, we find that reentrance is appreciable when the characteristic energy cost of the homogeneous and modulated phases are comparable to each other, and for systems in which the local order parameter is limited. In the asymptotic limit of high energy cost of the homogeneous phase we observe that the degree of reentrance decreases exponentially with the ratio of the characteristic energy cost of homogeneous and modulated phases. These mean-field results are confronted with Langevin simulations of an effective coarse grained model, confirming the expected extension of the reentrance in the phase diagram. These results shed new light on many systems undergoing inverse melting transitions by qualitatively improving the understanding of the interplay of entropy and energy around the inverse melting points.

## Introduction

The inverse melting (IM) is generally understood in terms of a larger entropic contribution arising when the systems go into a less-symmetric phase by increasing the temperature^1–5^. Several models have been used to reproduce such reentrant behaviors in particle systems^6^, spin systems^7–9^ and coarse grained approaches^10^. Even though in some of these systems the connection of the reentrant behavior with the specific features of the interactions and microscopic details has been clarified, there is a large class of systems with competing interactions in which such connection is yet to be established. Here we address the case of systems where isotropic competing interactions gives rise to modulated phases in two dimensions. Modulated phases typically appear in systems where a short-range attractive interaction competes with a non-local repulsive interaction. In two dimensions is commonly observed stripes and bubbles, or clusters, configurations with a modulation length depending on the relative strength of the interactions^11–14^. Systems with this kind of phenomenology are present from soft condensed matter to magnetic systems. For instance, in binary (AB) polymer mixture in which the polymer chains are connected by a covalent bond at the chain ends, microphase separation takes place. In this case, A-rich and B-rich domains can arrange them selves in lamelar structures of size of the order of the sum of each polymer chain^15–17^. In charged colloidal systems phase separation is possible due to the competition between the short range attractive Van der Waals interaction and the non-local repulsive screened Coulomb interaction^18–20^. On the other hand, ferromagnetic dipolar thin films present modulated structures due to the competition between the “attractive” exchange interaction and the “repulsive” dipolar interaction^21,22^.

In some of these systems the IM transitions have been predicted theoretically^23^ or even observed experimentally^24^. In the magnetic case, an IM transition has been recently observed in experiments on ultrathin ferromagnetic films with perpendicular anisotropy of Fe/Cu(001)^5,25,26^, which have shown that a perpendicular magnetic field versus temperature phase diagram displays strong reentrant features. The reentrance observed in this case occurs lowering temperature at constant field, characterizing an inverse melting process between a modulated phase and the uniform phase. These phases are analogous to crystalline and liquid phases, respectively. The behavior observed experimentally has been somewhat predicted theoretically for this particular magnetic systems^27^. In an attempt to explain this experimental observations, a scaling theory was developed to relate the presence of a certain family of microscopic interactions with the existence of reentrant behavior in the phase diagram^28^. More recently, the same reentrant behavior have been found within mean-field approximations for systems with dipolar repulsive interactions in the context of Landau-Ginzburg models^29^, lattice models^30^ and others^31^. Many of these efforts focus on the relation between the IM behavior with an entropy gain from domain wall degrees of freedom of the modulated patterns. In spite of these advances, little progress have been made relating the nature of the microscopic interactions and the underlying mechanisms of the IM transitions in the phase diagram.

In the present work we are able to explore this relation by generalizing a previously developed minimization-variational technique^31^ to consider generic isotropic interactions and different approximations schemes. Here we show that, in order to observe an IM transition, the relative energy cost of the homogeneous phase must comparable to the characteristic energy cost of the modulated phase. Furthermore, we found that another key ingredient for the IM transition to take place is that the local entropy has to be a steep enough function of the order parameter close to its saturation value. Physically, the steepness of the functional form of the local entropy establishes a constraint in the order parameter. In other words, we need that the order parameter has to be limited, not necessarily by a hard constraint. Finally, the overall key results are confirmed by Langevin simulations for the ferromagnetic dipolar model. To our knowledge, these are the first computer simulations to verify a clear reentrant behavior in systems with competing interactions.

## Results

Let us start by considering an Ising-like spin system {*s*_*i*_} with generic non-local interactions *A*_*ij*_ in a two dimensional square lattice. This Hamiltonian can be written in the form:

1 $$ {\mathcal H} =\frac{1}{2}\,\sum _{i,j}\,{A}_{ij}{s}_{i}{s}_{j}-\sum _{i}\,h{s}_{i},$$

where *h* is an applied external field. Within this general expression, we will focus on those interactions that are able to generate spatially modulated patterns in the equilibrium regime of the system.

Typically, these spatial textures are in form of stripes or bubbles patterns, which can extend over a large number of lattice sites. In dipolar frustrated ferromagnetic materials, for instance, the typical stripe width is about thousand times the lattice spacing^5^. On the other hand, theoretical and experimental studies on systems whose modulation is of the order of the minimum distances between their constituents have encounter no evidence of IM^32^. This scenario fully justify the use of coarse-grained approaches to study the IM transition.

After a coarse-graining extension of Eq. (1), the new effective 2D model is now described by a scalar-field local-order parameter $\varphi (\overrightarrow{x})$, with effective Hamiltonian written in the form^31,33^:

2 $$H[\varphi ]=\frac{1}{2}\,\iint \,{d}^{2}x{d}^{2}x^{\prime} \varphi (\overrightarrow{x})\varphi (\overrightarrow{x^{\prime} })A(|\overrightarrow{x}-\overrightarrow{x^{\prime} }|)+\frac{1}{\beta }\,\int \,{d}^{2}xS(\varphi (\overrightarrow{x}))-\int \,{d}^{2}xh\varphi (\overrightarrow{x}),$$

where the generic interaction $A(\overrightarrow{x},\overrightarrow{x}^{\prime} )=A(|\overrightarrow{x}-\overrightarrow{x}^{\prime} |)\equiv A(r)$ is considered isotropic, *β* stands for (*KT*)^−1^ and

3 $$S(x)=\frac{(1+x)}{2}\,\mathrm{ln}\,(\frac{1+x}{2})+\frac{(1-x)}{2}\,\mathrm{ln}\,(\frac{1-x}{2})$$

represent a local potential for the continuous order parameter, which can be seen as the microscopic entropic contribution to the coarse grained model. In fact, this form of the Hamiltonian is a local density approximation on the mean-field free energy of the model of Eq. (1).

In the following, we study the equilibrium phase diagrams of the Hamiltonian of Eq. (2). This is done by looking at the stationary states of the overdamped Langevin equation of the system in terms of the local order parameter $\varphi (x)$. Considering Eq. (2), a natural choice for the dynamical equation reads

4 $$\begin{array}{rcl}\frac{\partial \varphi (\overrightarrow{x},t)}{\partial t} & = & -\,\frac{\delta H[\varphi ]}{\delta \varphi (x)}+\eta (\overrightarrow{x},t)\\  & = & -\,\int \,{d}^{2}x^{\prime} A(|\overrightarrow{x}-\overrightarrow{x^{\prime} }|)\varphi (\overrightarrow{x^{\prime} },t)\\  &  & -\,\frac{1}{\beta }\,{\rm{arctanh}}(\varphi (\overrightarrow{x},t))+h+\eta (x,t),\end{array}$$

where $\eta (\overrightarrow{x},t)$ represent the usual zero-mean white noise, with $\langle \eta (\overrightarrow{x},t)\eta (\overrightarrow{x}^{\prime} ,t^{\prime} )\rangle =2{\beta }^{-1}\delta (t-t^{\prime} )\delta (\overrightarrow{x}-\overrightarrow{x}^{\prime} )$.

In the asymptotic regime such dynamical equation should describe the equilibrium state of the system. At a crude mean-field level, the mean-field free energy will coincide with Eq. (2). In general, more sophisticated approximation schemes will lead to different functional forms of the entropy functional. We have considered both mean-field and mean-field including local fluctuations schemes for the construction of the entropic contribution, as shown in the methods section.

### Free energy minimization

The minimization technique we perform in this work is analogous to that used in ref. ^31^. We are interested in solutions that minimize the effective free energy. We consider three types of solutions: (*i*) *stripes*, where $\varphi (\overrightarrow{x})$ is given by a one dimensional modulation; (*ii*) *bubbles*, where a two dimensional modulation occurs in the form of a triangular array of opposite values of $\varphi$ in relation to the background value; and (*iii*) *uniform*, where $\varphi$ takes a constant homogeneous value. Experiments consistently reveals that these are the local structures developed by these kind of systems. Whereas these are the basic local structures, at the large scales, spatial fluctuations break down their periodic and regular features. Nevertheless, at the mean-field level, this kind of fluctuations are not considered. Consequently the construction of the phase diagrams are made comparing the free energies of these three perfectly ordered solutions. All of these solutions can be written in general as:

5 $$\varphi (\overrightarrow{x})=\sum _{i=0}^{n}\,{c}_{i}\,\cos ({\overrightarrow{k}}_{i}\cdot \overrightarrow{x}),$$

where the set of wave vectors ${\overrightarrow{k}}_{i}$ are conveniently taken in order to reproduce the different solutions. For numerical purposes we have used 15 as the maximum number of modes along the principal direction, for each kind of solution. The wave vectors are defined as ${\overrightarrow{k}}_{i}={k}_{{\rm{eq}}}{\overrightarrow{a}}_{i}$, where the set of vectors ${\overrightarrow{a}}_{i}$ are chosen as a regular 1D (stripes) or 2D triangular (bubbles) lattice, of spacing equal to 1^31^.

Assuming a local order parameter in the form of Eq. (5), the functional Hamiltonians in Eqs (2) and (17) becomes then functions of *k*_eq_ and the set of amplitudes {*c*_*i*_}, for each kind of solution. Such functions are minimized to find the best parameters corresponding to the three type of solutions for a given point in the *H*-*T* space. The solution with minimal free energy is used to construct the *H*-*T* phase diagram.

This process is carried out for some representative interactions of this class of pattern forming systems $A(r)=\int \,\frac{{d}^{2}k}{{(2\pi )}^{2}}\hat{A}(k){e}^{i\overrightarrow{k}\cdot \overrightarrow{r}}$, where the function $\hat{A}(k)$ is the so called *fluctuation spectrum*. In order to develop modulated structures, $\hat{A}(k)$ must have a negative minimum at some nontrivial wave vector ${k}_{0}\ne 0$, resulting from the competitive nature of the interactions. A key quantity that is expected to control the relative stability of the uniform (homogeneous) phase versus the modulated phase is the ratio $\hat{A}(0)/|\hat{A}({k}_{0})|$. The value of $\hat{A}\mathrm{(0)}$ is largely influenced by the intensity and range of the repulsive interaction. In Table 1 we list the analytic forms of the fluctuation spectra considered in this work. As discussed above, these interactions correspond to several models physically relevant for condensed matter. The quadratic fluctuation spectrum has been extensively used to study different aspects of the Ising ferromagnet with dipolar interactions^34,35^. On the other hand, the quartic spectrum is the continuous limit of a system with competing first-neighbors attractive and second-neighbors repulsive interaction^36^, this kind of fluctuation spectrum have been used in effective models of diblock copolymers in the weak segregation limit^37,38^. We also consider a model where a short range attraction competes with a repulsive potential of Yukawa type, which has been used to model cluster forming colloidal systems^39^. In addition, we analyze the single mode case, which is a minimal prototype fluctuation spectrum of those considered here. For this interaction the restriction in the number of modes is included in the model by setting to infinity the energy cost of all but the zero and the principal modes. Though unrealistic, this model becomes a benchmark for our theoretical calculations.

**Table 1:** Analytical forms of several fluctuation spectra $\hat{A}(k)$ leading to modulated configurations, as are treated in this work, each corresponding to a physical system (see text).

Name	Expression	System
quadratic model	$\hat{A}(k)=a{(k-1)}^{2}-1$	ferromagnetic films^34,35^
quartic model	$\hat{A}(k)=a{({k}^{2}-1)}^{2}-1$	diblock copolymers^37,38^
screened Coulomb model	$\hat{A}(k)=-\,2(2+{a}^{2})+{k}^{2}+\tfrac{2{(1+{a}^{2})}^{3/2}}{\sqrt{{k}^{2}+{a}^{2}}}$	colloidal solutions^39^
single-mode approximation	$$\hat{A}(k)=\{\begin{array}{ll}{A}_{0} & {\rm{if}}\,k=0\\ -\,1 & {\rm{if}}\,k=1\\ +\,\infty & {\rm{elsewhere}}\end{array}$$	

In all cases the minimum has been set to $\hat{A}({k}_{0}=1)=-\,1$. The free parameter (*a*) is positive, and it is chosen in such a way that the control parameter $\hat{A}(0)$ has the same value for all models.

**Figure 1:**
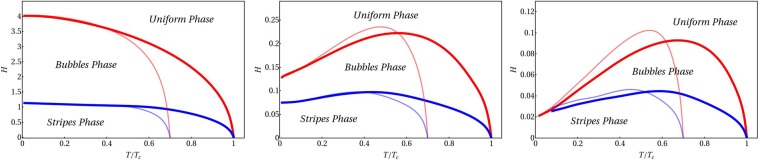
Phase diagrams corresponding to a system with fluctuation spectrum of the quadratic type, setting the number of modes in the principal directions to ${n}_{{\rm{\max }}}=15$. Different diagrams corresponds to different values of the fraction $\hat{A}\mathrm{(0)/|}\hat{A}({k}_{0})|$: *left* 4.0; *center* −0.6; *right* −0.8. Thick lines correspond to diagram lines calculated via mean field (2), while thinner lines are calculated with the improved mean-field minimization (17). Both temperature and external field are in units of *A*(*k*_0_).

Results for the phase diagrams of the quadratic model are shown in Fig. 1. Firstly, it is remarkable that improved mean field (thinner lines) and strict mean field approximations (thick lines) yield qualitatively similar phase diagrams. It is worth noting that the critical temperatures in the improved mean field case are lower than the mean field ones - as expected, since in the former case local fluctuations are included. Additionally, the values of the critical fields as a function of the reduced temperature are very close. Considering that the strict mean field approach has been used more often in previous works^31,33^, in what follows the overall presented analysis have been obtained within this approximation.

Interestingly, it can also be observed from Fig. 1 that the IM transition becomes significant as the quantity $\hat{A}(0)/|\hat{A}({k}_{0})|$ is decreased. For large values of $\hat{A}(0)/|\hat{A}({k}_{0})|$ (left panel), for a fixed value of the applied field, an increase of the temperature leads to an increase of the symmetry of the equilibrium configuration. However, a different phenomenology emerge in the center and right panels where the quantity $\hat{A}(0)/|\hat{A}({k}_{0})|$ takes values of −0.6 and −0.8. In these cases, the low temperature regime of the phase diagrams is characterized by an IM phase boundary, in which a transition between the homogeneous and modulated phases is observed as the temperature is increased.

### Reentrant behavior

We quantify the extension of the IM by defining the following reentrance parameter:

6 $$R=\frac{{h}_{{\rm{\max }}}-{h}_{0}}{{h}_{{\rm{\max }}}},$$

where ${h}_{0}={h}_{c}(0)$ is the critical field separating the modulated and homogeneous phase at zero temperature, while ${h}_{{\rm{\max }}}={h}_{c}({T}_{{\rm{\max }}})$ represent the maximum value of field along the phase boundary between these phases. The parameter in Eq. (6) is zero for non-reentrant diagrams, and takes the value 1 when ${h}_{0}=0$, when the IM transition is observed for all fields of the phase boundary between homogeneous and modulated phases.

**Figure 2:**
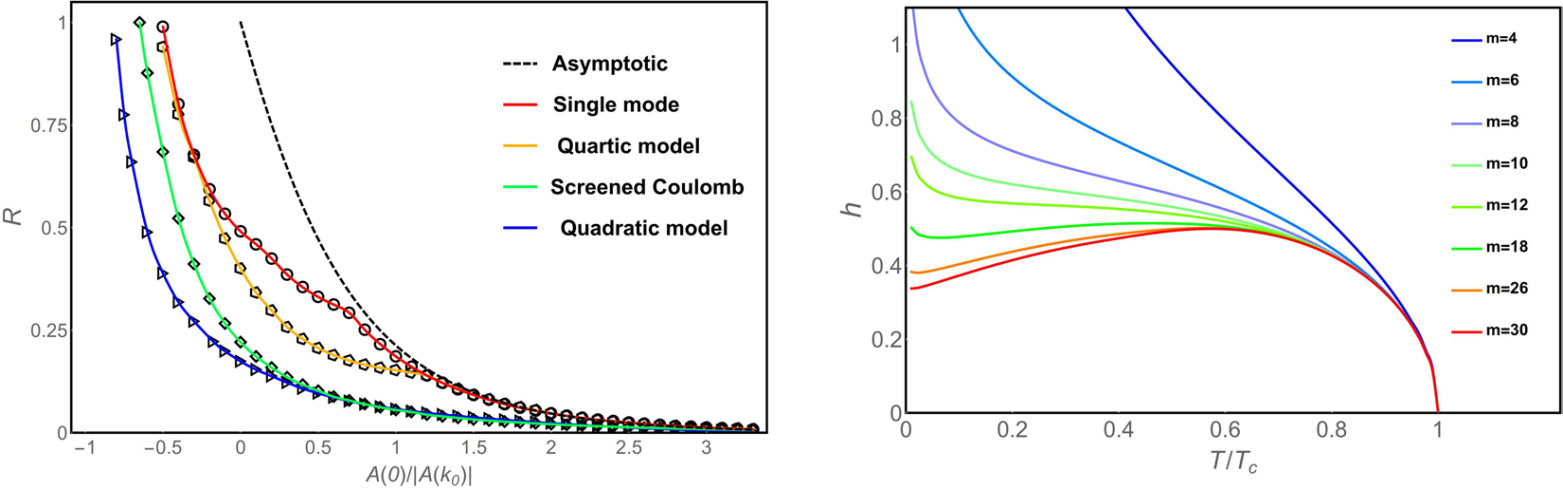
*Left*: Reentrant parameter as function of the relation $\hat{A}\mathrm{(0)/|}\hat{A}({k}_{0})|$. Solid curves correspond to the different interaction families presented in Table 1. The dashed line is the theoretical behavior valid in the asymptotic regime. *Right*: Critical line ${h}_{c}(T)$ for a system in the single-mode approximation with $\hat{A}(0)=0$, where the local potential has been expanded in a Taylor series up to *m* orders. Large values of *m* are needed for the system to display IM features; this translates into a steeper entropic functional near the saturation value of $\varphi$.

In Fig. 2 the reentrant parameter is shown for several families of interaction Hamiltonians. As can be seen, the previously observed behavior of the IM with respect to the relation $\hat{A}\mathrm{(0)/|}\hat{A}({k}_{0})|$ is confirmed for all types of interaction considered. In all cases, this relation is able to tune the reentrant properties of the systems in the whole range $0\le R\le 1$. This result suggest that the IM can emerge in systems with any particular type of competing interactions, as long as the relation $\hat{A}\mathrm{(0)/|}\hat{A}({k}_{0})|$ is sufficiently small. The generality of this conclusion should not be interpreted as a claim that IM is an ubiquitous phenomenon in such systems. Considering that $\hat{A}\mathrm{(0)/|}\hat{A}({k}_{0})|$ is proportional to the relative strength of the repulsive interaction, and that this is an intrinsic property of the system, to tune experimentally its value to the appropriate competing regime could be a difficult task. In fact, the narrow regime of $\hat{A}\mathrm{(0)/|}\hat{A}({k}_{0})|$ in which reentrance is appreciable could explain why this phenomenon is rarely observed.

It is also evident from Fig. 2 that, for a given value of the relation $\hat{A}\mathrm{(0)/|}\hat{A}({k}_{0})|$, the single-mode approximation presents the highest reentrance among all the different families. The reason of this finding relies in the fact that single-mode solutions implies a perfect sinusoidal structure. At small temperatures, the actual modulated solutions tend to be very structured, with important contribution of higher harmonics to lowering the free energy of the modulated solution. Consequently, the single mode ansatz possesses the highest free energy among the different modulated solutions, and thus, the lower critical field separating the modulated and homogeneous phases.

This difference of free energies/critical fields becomes less important as the temperature increases, since the weight of the higher harmonics decreases due to the effects of the entropic term in the free energy, regardless the specific form of the interactions. Eventually, in the vicinity of *T*_*c*_, the single mode solution is asymptotically exact. In summary, among all solutions, the single mode one has the lowest *h*_0_, while *h*_max_ is not particularly decreased. This explains why the single mode curve in Fig. 2 shows the highest reentrance for a given $\hat{A}\mathrm{(0)/|}\hat{A}({k}_{0})|$, and is an upper bound for the different interaction families given the values $\hat{A}\mathrm{(0)}$ and $\hat{A}({k}_{0})$. In this way, the relation $\hat{A}\mathrm{(0)/|}\hat{A}({k}_{0})|$ is the only needed input to know the maximum reentrance that any phase diagram can have.

The same arguments can be used to find a lower bound for the reentrance parameter of a given interaction $\hat{A}(k)$. Let us consider two fluctuation spectra ${\hat{A}}_{1}(k)$ and ${\hat{A}}_{2}(k)$, such that they have the same values of $\hat{A}\mathrm{(0)}$ and $\hat{A}({k}_{0})$, and ${\hat{A}}_{1}(k)\le {\hat{A}}_{2}(k)$ for all wave vectors. Then, ${\hat{A}}_{1}(k)$ will exhibit a lower free energy of the modulated solutions, due the contribution of higher harmonics. Again this difference is particularly important at lower temperatures, and decreases as temperature increases. This implies that ${\hat{A}}_{1}(k)$ has higher critical fields, *h*_*c*_(*T*), separating the modulated and homogeneous phases, but this difference increases as temperature decreases. Consequently, the reentrance parameter for ${\hat{A}}_{1}(k)$ will be smaller then that for ${\hat{A}}_{2}(k)$. Among all the interaction families considered in Table 1, the quadratic model has a fluctuation spectrum that limits inferiorly the other models (the quadratic model stands beneath the screened Coulomb model only when $\hat{A}\mathrm{(0)}\le 0$) and as a consequence, it presents the lowest reentrance parameter in Fig. 2.

### Low Reentrance Regime

As can be seen from Fig. 1, for large values of $\hat{A}\mathrm{(0)/|}\hat{A}({k}_{0})|$ the inverse transition appear just in a narrow region, corresponding to high external fields and low temperatures. In such conditions the transition between the modulated and the uniform phases is expected to occur with an average order parameter (magnetization) close to its saturation value. This implies that, close to the critical line, the modulations have small amplitude, which means that the transition should be continuous or, at most, weakly first order. Within this assumption an analytical study of the transition can be performed by using a single-mode description.

According to Eqs (5) and (13), the transition between the modulated (bubble) and the uniform phase, in the high $\hat{A}\mathrm{(0)/|}\hat{A}({k}_{0})|$ regime, must be governed by the effective free energy

7 $$H({c}_{0},{c}_{1})=\frac{1}{2}\hat{A}(0){c}_{0}^{2}-\frac{3}{4}|\hat{A}({k}_{0})|{c}_{1}^{2}+\frac{KT}{2}S({c}_{0})+KT\frac{3}{4}{S}^{(2)}({c}_{0}){c}_{1}^{2}-h{c}_{0},$$

where *c*_0_ is the spatial average of the order parameter (e.g. magnetization), and *c*_1_ is the amplitude of the modulation. Additionally, ${S}^{(n)}(\varphi )$ represent the derivative of order *n* of the function $S(\varphi )$. Performing the minimization process and enforcing the marginal stability of the modulated phase $\frac{{\partial }^{2}H}{\partial {c}_{1}^{2}}({c}_{0},{c}_{1})=0$, we obtain

8 $${c}_{0,c}(T)={S}_{-1}^{(2)}(\frac{|\hat{A}({k}_{0})|}{KT})$$

9 $${h}_{c}(T)=\hat{A}(0){c}_{0,c}+KT{S}^{(1)}({c}_{0,c}),$$

where ${S}_{-1}^{\mathrm{(2)}}(\varphi )$ represents the inverse function of ${S}^{\mathrm{(2)}}(\varphi )$. For our particular form of $S(\varphi )$ the final expression of the critical line *h*_*c*_(*T*), separating the modulated from the uniform phase, will be

10 $${h}_{c}(T)=\hat{A}(0)\sqrt{1-\frac{KT}{|\hat{A}({k}_{0})|}}+\frac{KT}{2}\,\mathrm{log}\,(\frac{1+\sqrt{1-\frac{KT}{|\hat{A}({k}_{0})|}}}{1-\sqrt{1-\frac{KT}{|\hat{A}({k}_{0})|}}}).$$

With the analytic form of the critical line it is possible then to obtain the behavior of the reentrant parameter in the asymptotic regime of large $\hat{A}\mathrm{(0)/|}\hat{A}({k}_{0})|$. According to its definition in Eq. (6) we have

11 $$R=\frac{2\,\exp \,(\,-\,1-\frac{\hat{A}(0)}{|\hat{A}({k}_{0})|})}{\frac{\hat{A}(0)}{|\hat{A}({k}_{0})|}+2\,\exp \,(\,-\,1-\frac{\hat{A}(0)}{|\hat{A}({k}_{0})|})}.$$

For comparison, together with the results obtained by direct minimization of the mean-field free energy, the asymptotic behavior analytically obtained in Eq. (11) is presented in Fig. 2 with dashed lines. As can be observed, the convergence to the asymptotic behavior occurs at relatively low values of $\hat{A}\mathrm{(0)/|}\hat{A}({k}_{0})|$ for the quartic model and the single-mode interactions. These are precisely the fluctuation spectra with the highest energy cost for modes beyond the principal one, making these systems to form nearly single-mode profiles, which is the approximation assumed in Eq. (7).

### Influence of the entropy functional

A careful analysis of the results obtained in Eqs (9) and (10) confirm that, within the regime of large $\hat{A}\mathrm{(0)/|}\hat{A}({k}_{0})|$, in order to produce a reentrant critical line, the entropic functional $S(\varphi )$ must be steep enough in the vicinity of the saturation value of the order parameter. To understand this result it is important to notice the role of each term in Eq. (9). The first term represents an energetic contribution proportional to the average value of the order parameter along the critical line, which is expected to increase towards saturation value as $T\to 0$ (see for example Eq. (10)). This term accounts for a typical behavior of ${h}_{c}(T)$, and cannot produce a reentrant critical line.

On the other hand, the second term of Eq. (9), which represents the entropic contribution, is proportional to the first derivative of the entropic function with respect to the order parameter, ${S}^{\mathrm{(1)}}({c}_{\mathrm{0,}c})$, evaluated at the average order parameter along the critical line. To develop a reentrant ${h}_{c}(T)$ close to zero temperature, it is necessary that ${S}^{\mathrm{(1)}}({c}_{\mathrm{0,}c}(T))$ to be large enough as ${c}_{\mathrm{0,}c}(T)$ approaches the saturation value in order to overcome the contribution of the first term in Eq. (9). In other words, the entropic function must be steep enough close to the saturation value of the order parameter in order to develop a reentrant behavior. It is worth recalling that even thought this result was obtained within certain approximations, still it suggests a central ingredient to observe IM in the considered models.

In order to clarify the role of this ingredient, we calculate the critical line ${h}_{c}(T)$ for successive approximations to the entropic function, systematically increasing its steepness near the saturation value. We focus in the single-mode case, where the reentrance is the largest, and fix the value $\hat{A}(0)=0$. We then expand the entropic function in a Taylor series up to order *m* around $\varphi =0$, where the larger the value of *m*, the steeper the entropic functional become around the saturation values $\varphi =\pm \,1$. The critical lines ${h}_{c}(T)$ for values $4\le m\le 30$ are shown in Fig. 2, where we can see that a reentrant behavior appears above $m\approx 16$, and at $m\ge 30$ we can notice a reentrance similar to the ones observed in the experiments on ultrathin ferromagnetic films with perpendicular anisotropy of Fe/Cu(001)^5,25,26^.

Since the single-mode approximation is the case with highest reentrance for any $\hat{A}\mathrm{(0)/|}\hat{A}({k}_{0})|$, the value $m=30$ represents the order of magnitude of the minimum power of the expansion of $S(\varphi )$ that produces a reentrance in the whole low temperature regime. Even though this result is for the $\hat{A}(0)=0$ case, the same procedure could be extended to find a general result determining the minimal *m* or steepness that produces the kind of reentrance in consideration, independently of the value $\hat{A}\mathrm{(0)}$. The above discussions shows that the IM phenomenology is dependent, at the same time, on the relative energy cost of the homogeneous phase ($\hat{A}\mathrm{(0)/|}\hat{A}({k}_{0})|$, see Eq. (11) and Fig. 2), and on the steep nature of the local potential around the saturation value of the order parameter.

### Langevin simulations

In order to explore the extent of our theoretical results, it would be interesting to compare them with simulations. Indeed, these inverse transitions in the presence of external fields have been explored within microscopic models for dipolar frustrated ferromagnetic materials, namely the dipolar Ising model^32^ and the dipolar Heisenberg model with perpendicular anisotropy^29^. On the other hand, simulation of coarse-grained models^35,40–42^ has also been used as an alternative to study the long wavelength behavior of these models.

In general, all these numerical approaches have failed in reproducing the IM transition. This is mainly due to the lack of a clear understanding of the features that a model has to include in order to develop that kind of behavior. As discussed above, the effective model presented here in Eq. (4) contains all the necessary ingredients to perform the reentrant transition with the appropriate set of parameters. In the following, we will consider the quadratic model (see Table 1) with several values of the curvature *a*.

The simulations are performed by numerical integration of the Langevin equation Eq. (4). In contrast with the analytical treatment developed in the previous sections, the simulations consider all possible modes and fluctuations consistent with the system size. In this way, the simulation also constitutes an ultimate proof of the validity of the analytical results obtained above.

The three cases considered for the quadratic model have curvature $a=0.1$, 0.4 and 5.0, corresponding to $\hat{A}(0)/|\hat{A}({k}_{0})|=-\,0.9$, −0.6 and 4.0, respectively (see Table 1). Simulation details are described in the methods section.

**Figure 3:**
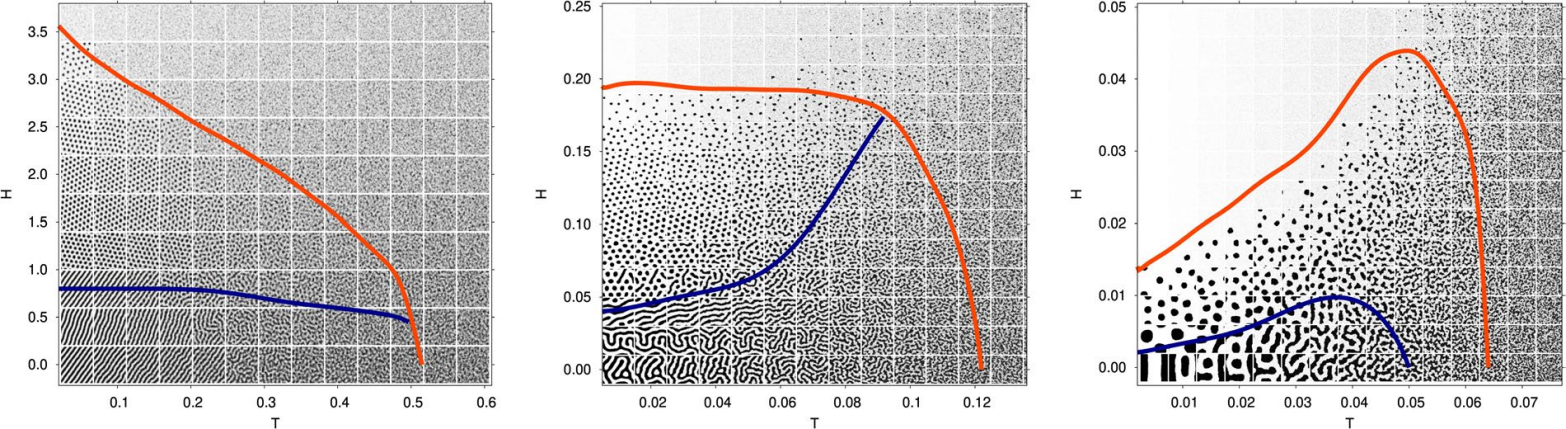
Phase diagrams obtained by extensive Langevin simulations of a system interacting via a quadratic fluctuation spectrum. The left panel correspond to $\hat{A}(0)/|\hat{A}({k}_{0})|=4.0$, followed by $\hat{A}(0)/|\hat{A}({k}_{0})|=-\,0.6$ and $\hat{A}(0)/|\hat{A}({k}_{0})|=-\,0.9$. Configurations indicate the representative state of $\varphi (\overrightarrow{x})$ in gray scale, ranging from −1 (black) to +1 (white). The center of the picture of the configurations corresponds to its (*T*, *H*) value in the phase diagram. The actual resolution of points explored in the simulations is finer, and was used to estimate the modulated/non-modulated (red) and stripe/bubble (blue) separation lines (see text). Notice that both these criteria does not distinguish the degree of order, so the lines do not correspond necessarily to phase transitions.

In order to identify the region where modulation sets in, we have estimated the $\langle \varphi (0)\varphi (\overrightarrow{x})\rangle$ correlation length through a nonlinear fit of the circularly averaged structure factor to ${\hat{S}}^{-1}(k)=b{(k-{k}_{0})}^{2}+r$, so that the correlation length is given by $\xi =\sqrt{b/r}$. Thus, the modulated region of the phase diagram is defined here as the region in which the correlation length of the system is larger than half of the basic modulation length. This estimate of the crossover between modulated and homogeneous/non-modulated regions is depicted in Fig. 3 with a red line.

In addition to the modulated region, it is interesting to differentiate regions dominated by bubbles or stripes. This can be done quantitatively by measuring the area *A* and perimeter *L* defined by the $\varphi (\overrightarrow{x})=\langle \varphi \rangle$ closed contours. Controlling the averaged value of the quantity $4\pi A/{L}^{2}$ (which is 1 for a circle, and formally 0 for an infinite stripe), if its resulting value is greater than $\frac{1}{2}$, then we consider that the configurations are dominated by bubbles. Otherwise, they are dominated by stripes. This estimate of the crossover between stripe dominated and bubble dominated modulation patterns is depicted in each case in Fig. 3 with a blue line. As one can notice, the distinction between these two basic textures is clear at low temperatures, while at moderate and high temperatures the boundary depends strongly on the chosen threshold value for $4\pi A/{L}^{2}$.

In Fig. 3 the phase diagrams obtained by the Langevin simulations are presented for several values of the ratio $\hat{A}\mathrm{(0)/|}\hat{A}({k}_{0})|$. As can be seen, the IM is encountered in the simulation and its behavior with respect to the specific value of $\hat{A}\mathrm{(0)}$ is virtually the same as that predicted with the analytical approximations in Fig. 1. That is, smaller values of $\hat{A}\mathrm{(0)/|}\hat{A}({k}_{0})|$ imply larger reentrance. These results are a further support to the validity of the analytical outcomes.

For small temperatures the values of the critical fields observed in simulations and mean-field approach are very similar. For higher temperatures, however, the increasing role of the fluctuations makes the critical fields obtained in the simulations to remain below its mean-field counterparts. Consequently, the extent of the reentrance observed in the simulations is smaller than that observed within the mean-field approximations. Furthermore, thermal fluctuations strongly decrease the temperature window in which the modulated phases appear in comparison with the one expected in the mean field approximations. It is important to stress out that while in the mean-field diagrams the lines corresponds to phase transitions, here it is not necessarily the case. For example, there is no symmetry breaking taking place between a disordered modulated phase and a homogeneous phase.

## Discussion

In this report we have addressed the problem of the IM transition in the frame of a coarse-grained model with generic interactions. A detailed characterization of the different phase diagrams was obtained by means of a minimization-variational technique and the use of the mean-field form for the local entropic contribution. We identified two fundamental ingredients for the IM to take place. First, it is necessary a low enough energy cost of the homogeneous phase, relatively to the modulated phase. This is achieved whenever the non-local repulsive interaction is much weaker than the attractive local interaction. The second ingredient is that the local order parameter must be limited. This can be achieved either naturally, in systems where the microscopic variables are intrinsically limited, like spins in a magnetic material, or effectively, like in a fluid where density can be limited due to the presence of a hard-core potential.

Furthermore, the microscopic mechanism behind the reentrant behavior is closely related to these ingredients. For fixed external fields, as we increase temperature, the modulated solution undergoes a natural processes of softening that diminishes the regions in which the order parameter is close to the saturation value. This in turn can produce a significant entropy gain if the local entropy function is steep enough close to the saturation value. Whenever the free energy loss associated to this mechanism is high enough, the system undergoes a IM transition from the homogeneous to the modulated phase, as we increase temperature.

Now is left to understand why reentrance diminishes monotonically as $\hat{A}\mathrm{(0)}$ is increased, as can be observed in Fig. 2. As expected when $\hat{A}\mathrm{(0)}$ is increased there is an increasing energy cost of the homogeneous phase. Consequently, for small temperatures the critical fields separating the homogeneous and modulated phases ${h}_{c}(T)$ become larger, and a higher external field is needed to reach the homogeneous configuration. In the phase diagram region in which the IM occurs, this implies a larger magnetization, closer to the saturation value. Due to the interaction with the external field, there is an increasing importance of the energetic contribution to the free energy. As the role of the energy in the low-temperature and high external field region of the phase diagram is enhanced, the eventual free energy loss by the entropic mechanism in the onset of the modulated state, increasing temperature, is progressively less important. As a consequence, there is a monotonous narrowing of the field range in which the IM occurs with the increase of $\hat{A}\mathrm{(0)}$, leading to the smaller values of *R* observed in Fig. 2.

It is worth mentioning that our results are in agreement with previous works, in particular our conclusion that the reentrance parameter decays exponentially with the ratio $\hat{A}(0)/|\hat{A}({k}_{0})|$ explains why, when repulsive interactions of the form *r*^−*α*^ are present, the IM does not occurs if *α* is lower than the system’s dimension^28^. In this case it is simple to show that $\hat{A}(0)=+\,\infty$, which immediately justify the previous conclusion. Regarding the mechanism behind the IM, some authors^30,31^ have pointed out that when IM takes place, there is a significant increase of the domain wall width, as temperature is raised at constant field. This variation of the domain wall width is reflected in an entropy gain which ultimately justify the reentrance to a modulated phase. In the present work we have generalized these previous results by understanding that the entropy gain takes place mainly due the shrink of the saturated regions in the modulation profile, as temperature increases. This explain the appearance of IM not only in the previously studied cases but also in the single mode phase diagram, where IM takes place without changing the domain wall width.

The robustness of our findings were tested by considering different models, all of them showing excellent agreement with our general predictions. In the limit of large $\hat{A}\mathrm{(0)/|}\hat{A}({k}_{0})|$, the different models presents a similarly small extension of the IM in the phase diagram, showing good agreement with our analytical approximation. For arbitrary isotropic competing interactions, we have also found a lower and an upper bound to the extension of the IM in the phase diagram. For fixed values of *k*_0_, $\hat{A}({k}_{0})$ and $\hat{A}\mathrm{(0)}$, the reentrance associated to an arbitrary fluctuation spectrum will be smaller than the corresponding in the single-mode approximation, and larger than that of a quadratic model, whenever this is a lower envelope for the fluctuation spectrum.

As a further validation of the minimization-variational technique, a numerical scheme was developed for the Langevin equation of this system and the simulation results qualitatively confirm the main claims regarding the shape of the phase diagrams. For the dipolar system, our mean-field results show an appreciable reentrance for $\hat{A}(0)/|\hat{A}({k}_{0})| < 0$, while the simulations indicate $\hat{A}(0)/|\hat{A}({k}_{0})|\lesssim -\,0.6$. Indeed, the value of $\hat{A}\mathrm{(0)/|}\hat{A}({k}_{0})|$ below which the reentrance becomes appreciable in the mean field calculations is always an upper bound to observe reentrance in simulations or experiments.

Another interesting difference between simulations and mean field results is the boundary between bubble and stripe phases at moderate and high temperatures in Figs 1 and 3. This is expected, since the topological excitations of the patterns, phase coexistence and fluctuations present in simulations, in general cannot be accounted properly in a mean field description, especially in the high temperature region. Indeed, the numerical phase diagrams show a number of different phases that are beyond the mean-field solutions and beyond a simple distinction between stripe and bubble dominated patterns. The nature of these phases can only be tackled analyzing properly the topologial structures of the patterns. In this sense, the construction of a more detailed phase diagram will be addressed in a future work.

## Methods

### Mean field approximation

Within the mean field approximation, the stationary state of Eq. (4) implies that ${\partial }_{t}\langle \varphi (\overrightarrow{x},t)\rangle$ must be zero. Angular brackets indicate the thermal average of the corresponding quantity. Taking the thermal average over both sides of Eq. (4) we obtain

12 $$\frac{1}{\beta }\,\langle {\rm{arctanh}}(\varphi (\overrightarrow{x},t))\rangle =-\,\int \,{d}^{2}x^{\prime} A(|\overrightarrow{x}-\overrightarrow{x^{\prime} }|)\langle \varphi (\overrightarrow{x^{\prime} },t)\rangle +h.$$

At this point a relation between $\langle {\rm{arctanh}}(\varphi (\overrightarrow{x},t))\rangle$ and $\langle \varphi (\overrightarrow{x},t)\rangle$ is necessary, and different approximations could be used^33^. In the present work, we begin by using the standard mean-field approach, neglecting all fluctuations in the local order parameter: $\langle {\rm{arctanh}}(\varphi (\overrightarrow{x},t))\rangle \cong {\rm{arctanh}}(\langle \varphi (\overrightarrow{x})\rangle )$. In this case we can recast Eq. (12) as:

13 $$\frac{\delta H[\langle \varphi \rangle ]}{\delta \langle \varphi (x)\rangle }=0.$$

This means that, in the crude mean field approach, the equilibrium state of the system corresponds to configurations minimizing the effective coarse grained Hamiltonian $H[\varphi ]$, i.e. the mean field free energy of the original microscopic model of Eq. (1).

In order to test the robustness of the phase diagrams obtained within this approximation, we additionally calculate an improved version of this mean-field approach. This is accomplished by including the equilibrium non-linear dynamics of the local order parameter in Eq. (4). Let us then consider the exact Langevin equation of motion for a single site of the system. According to Eq. (4) we can write:

14 $$\frac{\partial \varphi (\overrightarrow{x},t)}{\partial t}={h}_{l}(\overrightarrow{x},t)-\frac{1}{\beta }\,{\rm{arctanh}}(\varphi (\overrightarrow{x},t))+\eta (\overrightarrow{x},t),$$

where ${h}_{l}(\overrightarrow{x},t)$ represents the local field in the equilibrium state. An improved mean field approximation can be devised by neglecting fluctuations in the local field. In this approximation the local field is simply taken as a constant along time, equal to the mean local field ${h}_{{\rm{eff}}}(\overrightarrow{x})\equiv \langle {h}_{l}(\overrightarrow{x},t)\rangle$. Once that we have decoupled the multiple single site equations, we can solve exactly the stochastic equation for generic single site. The single site Langevin equation resulting from Eq. (14) has a stationary equilibrium state satisfying the Boltzmann probability distribution, thus

15 $$P(\varphi )=\frac{1}{Z}\,\exp [\,-\,\beta (\,-\,{h}_{{\rm{eff}}}\varphi +\frac{1}{\beta }S(\varphi ))],$$

where *Z* is a normalization constant.

Once the form of the probability distribution $P(\varphi )$ is known, it is possible to calculate $\langle {\rm{arctanh}}[\varphi ]\rangle$ and $\langle \varphi \rangle$ as a function of the parameter $\beta {h}_{{\rm{eff}}}$. This allow to parametrically obtain the function $\langle {\rm{arctanh}}\rangle (\langle \varphi \rangle )$. In turn, this relation ($\langle {\rm{arctanh}}\rangle (\langle \varphi \rangle )$) can be used in Eq. (12) to obtain the improved mean-field description of the model. Interestingly, the final equation can still be written in the same form:

16 $$\frac{\delta {H}_{1}[\langle \varphi \rangle ]}{\delta \langle \varphi (x)\rangle }=0,$$

using the improved version of the Hamiltonian:

17 $$\begin{array}{rcl}{H}_{1}[\varphi ] & = & \frac{1}{2}\,\iint \,{d}^{2}x{d}^{2}x^{\prime} \varphi (\overrightarrow{x})\varphi (\overrightarrow{x^{\prime} })A(|\overrightarrow{x}-\overrightarrow{x^{\prime} }|)\\  &  & +\,\frac{1}{\beta }\,\int \,{d}^{2}x\,{S}_{1}(\varphi (\overrightarrow{x}))-\int \,{d}^{2}x\,h\varphi (\overrightarrow{x}),\end{array}$$

with the effective entropic contribution:

18 $${S}_{1}(\varphi )=-\,{\int }_{\varphi }^{1}\,dx\,\langle {\rm{arctanh}}\rangle (x).$$

Again, this means that the equilibrium state can be obtained by minimizing certain free energy functional. As can be seen, once we include the nonlinear local dynamics there is an entropy gain due to the soft nature of the order parameter - it is worth noting that local entropy is defined as $-\,S(\varphi )$ in Eqs (2 and 17). Once these free energy functionals of Eqs (3 and 18) have been defined, we can proceed with the minimization process to study the equilibrium states of the system.

### Langevin simulations

The Langevin equation of motion for the effective model can be written in the form

19 $$\frac{\partial \varphi (\overrightarrow{x},t)}{\partial t}=T\,{\rm{atanh}}\{\varphi (\overrightarrow{x},t)\}+H-{[A(k)\hat{\varphi }(\overrightarrow{k},t)]}_{\overrightarrow{x}}^{FT}+\eta (\overrightarrow{x},t)$$

where ${]}_{\overrightarrow{x}}^{FT}$ means the $\overrightarrow{x}$ component of the corresponding Fourier transform. In order to properly deal with the stiffness of Eq. (19), due to the first term in the r.h.s., we implement the following fully implicit first order scheme:

20 $$\begin{array}{rcl}\varphi (\overrightarrow{x},t+dt) & = & \tanh \{\,-\,\frac{\varphi (\overrightarrow{x},t+dt)}{Tdt}+\frac{1}{T}(\varphi (\overrightarrow{x},t)+H\\  &  & -\,{[A(k)\hat{\varphi }(\overrightarrow{k},t)]}_{\overrightarrow{x}}^{FT}+\eta (\overrightarrow{x},t))\}\end{array}$$

The above equation is discretized in a square lattice and solved on each site by the Halley’s method^43^. Lattice constant is chosen to be $dx=\pi /7$, so that the basic modulation length spans 14 lattice sites. The linear size is $L=112$, such that the system is able to accommodate 8 basic modulation lengths.

The three cases considered for the quadratic model have curvature $a=0.1$, 0.4 and 5.0, corresponding to $\hat{A}(0)/|\hat{A}({k}_{0})|=-\,0.9$, −0.6 and 4.0. The time steps used in Eq. (20) for each case are $dt=0.1$, 0.05 and 0.005, respectively. The estimated equilibration times are between 10^5^ and 10^7^ time steps for the $a=0.1$ case, and between 10^4^ and 10^6^ time steps for the $a=0.4$ and 5.0 cases. The phase diagrams were constructed by slow cooling protocol at constant external fields.
